# Iron Deficiency Promotes the Lack of Photosynthetic Cytochrome *c*_550_ and Affects the Binding of the Luminal Extrinsic Subunits to Photosystem II in the Diatom *Phaeodactylum tricornutum*

**DOI:** 10.3390/ijms232012138

**Published:** 2022-10-12

**Authors:** Carmen Castell, Encarnación Díaz-Santos, Luis G. Heredia-Martínez, Luis López-Maury, José M. Ortega, José A. Navarro, Mercedes Roncel, Manuel Hervás

**Affiliations:** Instituto de Bioquímica Vegetal y Fotosíntesis, cicCartuja, Universidad de Sevilla and CSIC, 41092 Seville, Spain

**Keywords:** photosystem II, cytochrome *c*_550_, PsbV, electron transfer, iron limitation, pulse-amplitude modulation chlorophyll fluorescence, thermoluminescence, *Phaeodactylum tricornutum*

## Abstract

In the diatom *Phaeodactylum tricornutum*, iron limitation promotes a decrease in the content of photosystem II, as determined by measurements of oxygen-evolving activity, thermoluminescence, chlorophyll fluorescence analyses and protein quantification methods. Thermoluminescence experiments also indicate that iron limitation induces subtle changes in the energetics of the recombination reaction between reduced Q_B_ and the S_2_/S_3_ states of the water-splitting machinery. However, electron transfer from Q_A_ to Q_B_, involving non-heme iron, seems not to be significantly inhibited. Moreover, iron deficiency promotes a severe decrease in the content of the extrinsic PsbV/cytochrome *c*_550_ subunit of photosystem II, which appears in eukaryotic algae from the red photosynthetic lineage (including diatoms) but is absent in green algae and plants. The decline in the content of cytochrome *c*_550_ under iron-limiting conditions is accompanied by a decrease in the binding of this protein to photosystem II, and also of the extrinsic PsbO subunit. We propose that the lack of cytochrome *c*_550_, induced by iron deficiency, specifically affects the binding of other extrinsic subunits of photosystem II, as previously described in cyanobacterial PsbV mutants.

## 1. Introduction

Photosynthetic organisms have additional iron requirements, as this metal is an indispensable cofactor in the photosynthetic electron transfer (ET) chain [1,2,3]. Consequently, the growth of photosynthetic algae—and of diatoms in particular—is limited by the availability of iron [2,3,4,5]. Moreover, most diatoms have extra iron requirements, as they have an extra heme protein, PsbV or cytochrome *c*_550_ (Cc_550_), associated with photosystem II (PSII); they also produce cytochrome *c*_6_ (Cc_6_) as the only electron carrier between the cytochrome *b*_6_*f* (Cb_6_f) and photosystem I (PSI) complexes [6]. This is in contrast to the situation in cyanobacteria and green algae, in which plastocyanin (a copper protein) can alternatively substitute Cc_6_ [1,6]. A down-regulation under iron limitation of several iron-containing photosynthetic proteins was previously reported in diatoms [7,8]. This includes, in particular, the iron-rich Cb_6_f and PSI complexes, but also Cc_6_ and ferredoxin (replaced by flavodoxin) [2,7,8,9]). However, although iron deficiency is considered to particularly affect PSI, and consequently decreases the PSI:PSII ratio, reduced photochemical rates and contents of PSII were also described in diatoms under iron-limiting conditions [4,8,9,10]. Besides photoinhibition and ET blocking processes, iron deficiency could disturb PSII activity by affecting either of the two iron components of the transmembrane intrinsic PSII core: the cytochrome *b*_559_ (Cb_559_) and the non-heme iron cofactor [11]. Whereas the first protein is strictly required for the assembly of PSII [12], the lack of the non-heme iron cofactor promotes the inhibition of the electron flow between Q_A_ and Q_B_ [13,14].

The PSII complex also comprises several extrinsic proteins which vary in the different groups of photosynthetic organisms [11,15,16,17]. In cyanobacteria, three extrinsic proteins are associated at the luminal side of PSII in the vicinity of the Mn_4_CaO_5_ cluster. These are PsbU, PsbO and PsbV/Cc_550_ [11,18]. Cc_550_ is a heme-protein with a three-dimensional structure similar to that of class I *c*-type cytochromes but exhibiting an unusual bis-histidine axial coordination and a hydrophobic extension in the C-terminal region that acts by anchoring the protein to the PSII core [19]. Cc_550_ also appears in eukaryotic algae from the red photosynthetic lineage, including diatoms [16,17,20,21,22], but is absent in the green lineage, which comprises green algae and plants, where it seems to be replaced by the non-iron-containing PsbP subunit [19,20,23]. In addition to Cc_550_, the PSII of diatoms contains another four luminal extrinsic proteins: PsbO, PsbU, PsbQ′ and Psb31 [17,23,24]. PsbO and PsbU bind to the same positions as the equivalent subunits in cyanobacterial PSII, whereas PsbQ′ binds to the same position as in red algae (and PsbQ in plant PSII) [17,21,24]. The solved PSII structure from the centric diatom *Chaetoceros gracilis* (*C. gracilis*) showed that Cc_550_ binds directly to the PSII core in the proximity of the D1 and CP43 proteins, close to the oxygen-evolving complex and in the same position as in cyanobacteria [11,17,21]. The Psb31 subunit binds directly to the intrinsic proteins of PSII, and could mitigate the chloride requirement for oxygen evolution and participate in the transfer of protons from the Mn_4_CaO_5_ cluster to the luminal surface [16,17].

The main role of Cc_550_ in PSII seems to be the stabilization of the Mn_4_CaO_5_ cluster, mainly through the binding of Cl^−^ and Ca^2+^ ions and facilitating the binding of the other extrinsic subunits [16,19,20,25,26,27,28,29]. Consequently, although it was demonstrated in cyanobacteria that Cc_550_ is not strictly required for PSII activity, its absence or functional mutation decrease PSII performance because it results in incomplete assembly of fully functional PSII complexes [27,29,30,31]. Another proposed function of Cc_550_ is that it contributes to entry/exit channels for water or protons from the Mn_4_CaO_5_ cluster in proton-coupled electron transfer via Y_z_ [17,18,29,32].

In this work, we further investigated the effects of iron deficiency on PSII activity in the model pennate diatom *Phaeodactylum tricornutum* (*P. tricornutum*). Moreover, the effects of iron limitation on the Cc_550_ levels were also explored, as it was previously reported that the Cc_550_ content decreases significantly when *P. tricornutum* cells grown in conditions of iron sufficiency are transferred to iron-limiting conditions [9,32].

## 2. Results

**Figure 1:**
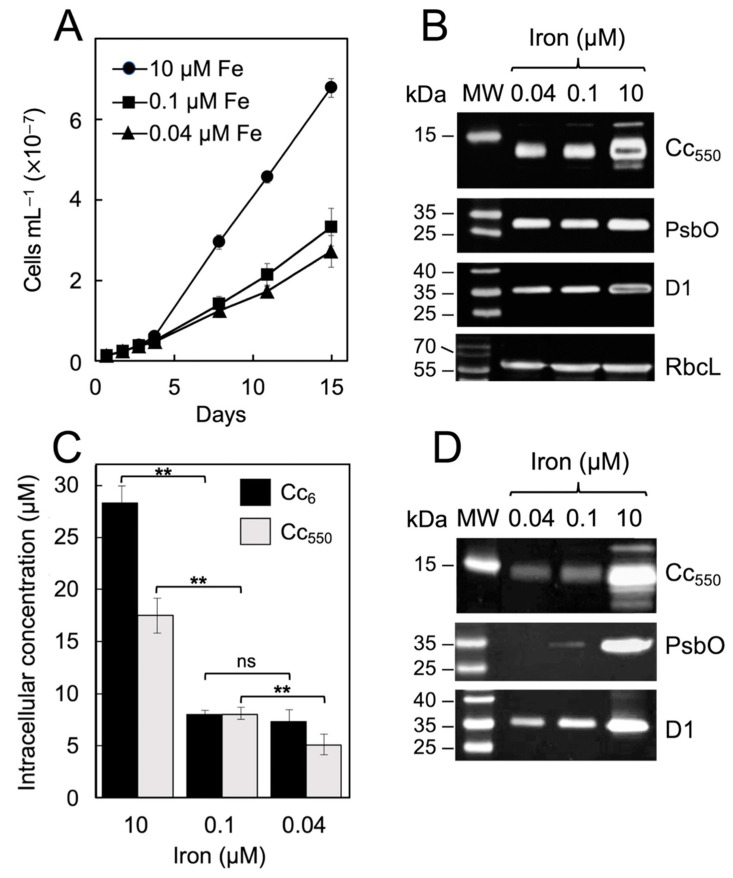
(**A**) Growth curves for *P. tricornutum* cultures under different iron concentrations. Mean values ± SD of measurements from 10 independent cultures are presented. (**B**,**D**) Representative Western-blots of different photosynthetic proteins in samples of whole cells (**B**) and membrane extracts (**D**) from *P. tricornutum* grown under iron-replete (10 µM iron) and iron-deficient (0.1 or 0.04 µM iron) conditions. MW: molecular weight standard; Cc_550_ and PsbO, the extrinsic cytochrome *c*_550_ and PsbO subunits of PSII; D1, the intrinsic D1 subunit of PSII; RbcL, control with the RuBisCO large subunit. Numbers on the left are the MW values corresponding to the molecular weight standard. Protein extracts: 20 µg for RuBisCO large subunit; 10 µg for PsbO and D1; 2.5 µg for Cc_550_. Membranes were incubated overnight with the following dilutions of the selected primary antibody: 1:1000 anti-Cc_550_ and anti-RuBisCO large; 1:2000 anti-PsbO; 1:10,000 anti-D1. The complete Western-blots are shown in Figure S1 (Supplementary Material). (**C**) Intracellular concentrations of cytochrome *c*_6_ (Cc_6_) and Cc_550_ measured by differential absorbance changes of *P. tricornutum* cells grown under different iron concentrations. Data represent mean values ± SD of at least four independent measurements. Double asterisks mark statistically different data groups (*p* < 0.01); ns = not statistically different.

We first analyzed the effect of different levels of iron availability on the growth of *P. tricornutum* cells under our experimental conditions. Three iron concentrations were selected: iron-replete (10 µM added iron), relatively mild iron-limiting conditions (0.1 µM iron) [9] and more severe iron limitation (0.04 µM iron). Figure 1A shows that, as expected, the growth of the cultures decreased as the amount of iron in the medium diminished, as well as the specific growth rates (Table 1) [7,33]. A more pronounced decrease in growth can be observed when lowering iron concentration from 10 to 0.1 µM, the variation then being less marked at an iron concentration of 0.04 µM (Figure 1A). 

To further characterize the effects on the PSII functioning induced by iron starvation in *P. tricornutum* cells, we analyzed different parameters related to the photosynthetic activity of this photosystem. First, the overall PSII activity was measured as the net photosynthetic oxygen evolution per cell, using 2,6-dichloro-1,4-benzoquinone (DCBQ) as the electron acceptor (Table 1). This allowed estimation of the PSII activity from water to the Q_B_ quinone site. As shown in Table 1, a decrease in the overall activity of PSII per cell is observed when the iron concentration decreases in the media. This decrease reaches levels of ≈40% of activity in the presence of 0.04 µM iron compared with the iron-replete condition (Table 1). In contrast, no negative effects of iron deficiency on the dark respiration activity were observed (Table 1).

**Figure 2:**
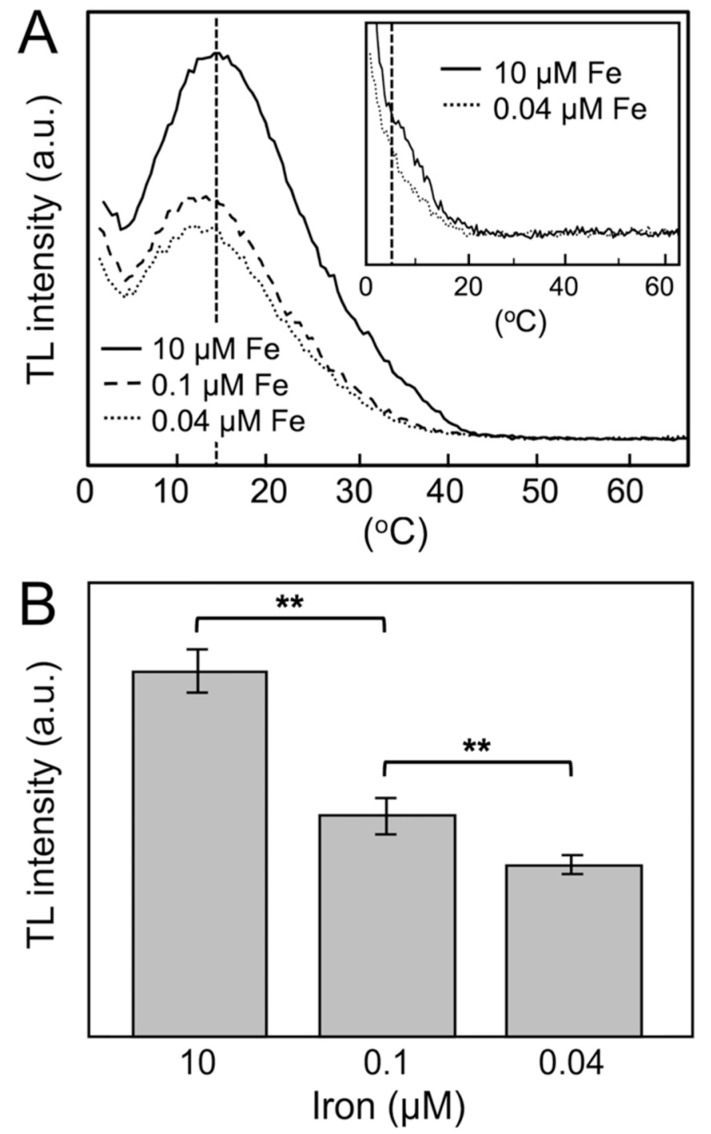
Effect of iron deficiency on the PSII activity of *P. tricornutum* cells measured by the TL technique. (**A**) Representative standard TL glow curves, obtained after excitation of *P. tricornutum* cells with two flashes at 1 °C under different iron concentrations. Inset: TL glow curve of *P. tricornutum* cells in the presence of 10 or 0.04 µM iron and 20 µM DCMU. (**B**) Intensities obtained from the component analysis of TL curves from six independent experiments (see also Table 1). Double asterisks mark statistically different data groups (*p* < 0.01). See the Methods and Materials section for more details.

The effect of iron deficiency on the ET PSII activity of *P. tricornutum* cells was further investigated using the standard thermoluminescence (TL) technique (Figure 2) [9,10,34]. Excitation of iron-deficient *P. tricornutum* WT cells with two flashes at 1 °C induced the appearance of typical TL glow curves, or the so-called B band, but with a significant decrease in the total signal intensity compared with iron-replete conditions (Figure 2 and Table 1) [9]. These TL signals arise from reverse ET recombination of reduced Q_B_^−^ with the S_2_/S_3_ states of the manganese cluster [35]. The deconvolution analysis of these emission curves enabled two components to be obtained, with *t*_max_ values of 16 and 29 °C, respectively, under iron-replete conditions (not shown). These components can be assigned to the well-known B1 and B2 TL bands, originating from the recombination reactions of S_3_Q_B_^−^ and S_2_Q_B_^−^ charge pairs, respectively [34,35]. The amplitude of the TL signal is thus related to the overall PSII activity from the water-splitting system to the final quinone acceptor [13,36]. The comparison between the conditions of iron sufficiency or limitation shows that the TL signal intensity and the net photosynthetic oxygen evolution per cell decrease in a parallel manner (Table 1). In the more severe iron-deficiency condition (0.04 µM iron), the TL signal drops to ≈47% of the values obtained under iron-replete conditions (Table 1), indicating a decrease in the number of active PSII centers [13]. 

Iron deficiency induces an additional effect on PSII centers with active ET, as demonstrated by a change in the *t*_max_ of the B1 and B2 TL bands. Thus, the *t*_max_ of the B1 band moves from 16 to 12 °C as the iron concentration decreases from 10 to 0.04 µM, whereas the *t*_max_ of the B2 band moves from 29 to 24 °C (not shown). These shifts indicate that the energy of the recombination reaction from Q_B_^−^ to the S_2_/S_3_ states is altered under iron-limiting conditions, probably due to subtle changes in the S states of the water-splitting machinery. Alternatively, the lack of iron could promote a limitation in the function of the non-heme iron cofactor in PSII, which acts as a redox mediator in the ET reaction from Q_A_ to Q_B_ [13]. In this case, the shifts of the TL B1 and B2 bands to lower temperatures would indicate the inhibition of Q_B_ reduction and, consequently, the appearance of TL signals arising from the recombination of S_2_Q_A_^−^ and S_3_Q_A_^−^ pairs (the so-called Q and A bands, respectively) [13,35]. However, experiments using 3-(3,4-dichlorophenyl)-1,1-dimethylurea (DCMU) seem to exclude this possibility (Figure 2A, Inset). DCMU acts by blocking ET from Q_A_ to Q_B_, thereby promoting the disappearance of the B bands and the emission of the S_2_Q_A_^−^ and S_3_Q_A_^−^ low-temperature Q and A bands (5 °C and −15 °C, respectively) [35]. Although these bands could not be fully resolved with our current TL setup, the addition of 20 µM of DCMU in iron-replete conditions promoted the absence of the B band and the appearance of a small shoulder at about 5 °C, arising from the S_2_Q_A_^−^ recombination Q band (Figure 2A, Inset). Equivalent but minor signals were obtained at 0.1 and 0.04 µM iron in the presence of DCMU. The non-appearance of the S_2_Q_A_^−^ band in the TL signal of experiments performed under iron limitation and in the absence of DCMU indicates that the low concentrations of iron used here do not promote a significant inhibition of ET from Q_A_ to Q_B_.

The relative content of PSII in whole cells was previously estimated on the basis of the total yield of variable chlorophyll *a* fluorescence (*F*_v_ = *F*_m_ − *F*_0_), in samples previously incubated in dark with *p*-benzoquinone and ferricyanide to oxidize any residual Q_A_^−^, and after the addition of DCMU and hydroxylamine (HA) [37,38]. As noted above, DCMU acts by blocking forward ET from Q_A_ to Q_B_, whereas HA acts as an electron donor for Y_z_^+^. Under these conditions, a saturating flash ensures that each PSII center reaches the high fluorescence yield state, thus providing a good biophysical measure of the relative PSII concentration that compares well with biochemical methods [37,38]. These fluorescence measurements are mainly governed by the redox states of Q_A_ and P_680_ at PSII [37] and, consequently, the estimated PSII content is related to the photochemically active PSII, capable of charge separation (from P_680_ to Q_A_). As indicated in Table 1, the relative content of photochemically active PSII per cell shows an iron-dependent decrease, reaching a value of ≈55% at 0.04 µM iron compared with iron-replete conditions. These values are consistent with the global activities observed both by photosynthetic oxygen evolution and TL experiments under the same conditions (Table 1). The maximum quantum yield of PSII, *F*_v_/*F*_m_, also decreases in iron-deficient cells in comparison with iron-replete cultures, although slightly higher values are obtained (≈63% at 0.04 µM iron; Table 1) [10,33]. 

A decrease in the PSII activity under iron limitation could be the consequence of either a lower amount of functional PSII complexes per cell and/or a lower PSII photochemical overall efficiency. Consequently, the levels of several PSII protein components were determined by Western-blot in *P. tricornutum* cells after 15 days of growth at the different iron concentrations, which specifically included the D1 intrinsic core protein and the extrinsic PsbO and PsbV/Cc_550_ subunits (Figure 1B). A reduction in the content of the D1, Cc_550_ and PsbO subunits was found when lowering iron concentrations, although this decrease appeared to be more pronounced in the case of Cc_550_ (Figure 1B). Western-blot bands were approximately quantified by using a specific analysis software (Quantity One^®^ 1-D analysis software, version 4.6.2, from Bio-Rad), and levels of ≈46% of D1 and ≈60% PsbO were roughly estimated at 0.04 µM iron compared with iron-rich conditions. The observed D1 levels are thus in agreement with the global activity observed both by photosynthetic oxygen evolution and TL under the same conditions (Table 1).

The changes in the amount of the Cc_550_ holoprotein can be determined more precisely by measuring the differential absorbance spectra of cell fractions (Figure 1C) [9,22]. A similar approach can be used to measure the levels of Cc_6_ and thus analyze the effects of iron deprivation on the content of both photosynthetic cytochromes. The obtained results (Figure 1C) confirm that iron deficiency drastically affects the relative contents of both Cc_550_ and Cc_6_ [9,10,22], which declined as the amount of iron in the cultures decreased. In addition, in the case of Cc_550_, spectrophotometric measurements show results equivalent to the Western-blot analysis (Figure 1B,C). Cultures grown under the lowest iron availability (0.04 µM iron) show levels of Cc_6_ and Cc_550_ of ≈25 and 28%, respectively, compared with iron-replete conditions (10 µM iron) (Figure 1C). A more pronounced decrease was noticed when lowering iron concentration from 10 to 0.1 µM iron, whereas the variation was less marked at the lowest iron concentration (Figure 1C), in which both cytochromes reached an equivalent intracellular level (5–7 µM) (Figure 1C). Thus, the concentrations of D1 and PsbO under the more restricted iron limitation (see above) are about two times higher compared with Cc_550_ levels relative to the iron-replete condition. 

The lack of Cc_550_ detected at lower iron concentrations could be accompanied by a decrease in the levels of other extrinsic proteins bound to PSII, such as the PsbO subunit, as previously described in cyanobacteria [29]. Consequently, we also investigated the content of D1 and the extrinsic PsbV/Cc_550_ and PsbO subunits bound to membrane fractions extracted from *P. tricornutum* cells under different iron availability. As shown in Figure 1D, as iron concentration decreases, the content in D1 also decreases, in a similar manner to that previously observed in whole cells. In contrast, the levels of Cc_550_ and PsbO were drastically diminished in the membrane extracts compared with whole cells (Figure 1B,D). However, whereas the decrease in Cc_550_ in the membrane fraction can be explained by the greater drop of this protein detected under iron-deplete conditions (and the existence of a free soluble population not bound to PSII [22,30]), the PsbO decay indicates a lower affinity of this protein for the binding to PSII at low iron concentrations. From these results, we deduce that the lack of Cc_550_ under iron-limiting conditions promotes a decreased binding of PsbO (and the other extrinsic subunits) to the PSII core and, therefore, the assembly of a number of PSII complexes affected in the water-splitting activity.

## 3. Discussion

Among other effects, iron starvation causes drastic alterations in the photosynthetic efficiency in diatoms, promoting a decrease in the content of both the iron-rich PSI and Cb_6_f complexes, resulting in an increased PSII:PSI ratio [7,8,39]. However, previous results in diatoms also indicated the occurrence of lower photochemical efficiencies and contents of PSII under iron-limiting conditions [4,8,9,10]. In addition, a drastic decrease in Cc_550_ (and Cc_6_) content was reported when cells cultured under conditions of iron sufficiency (12 µM iron) were transferred to relatively mild iron-limiting conditions (0.12 µM iron) [9,10,11,12,13,14,15,16,17,18,19,20,21,22]. In this work, we extended this preliminary study to analyze in depth the effects of iron deprivation on PSII function and the Cc_550_ content in *P. tricornutum*, in the latter case compared to changes in Cc_6_, the other luminal photosynthetic cytochrome.

Our results first indicate that iron limitation induces a significant decrease in the total content and activity of PSII in transferring electrons from water to quinones, as well as subtle changes in the S states of the water-splitting machinery. Moreover, a similar process of iron-induced downregulation occurs for the two luminal heme proteins, Cc_550_ and Cc_6_, under iron limitation. The decline in the content of Cc_550_ under iron-limiting conditions (0.04 µM iron), to ≈28% of the values observed under iron-replete conditions, contrasts with a decrease only to ≈45 and 60% in the content of D1 and PsbO, respectively. Although a decrease in the content of D1 can be associated with the observed overall decrease in the content of active PSII centers, our data indicate that Cc_550_ undergoes an additional drop related to a decrease in the iron levels. Thus, it seems that in the framework of a general effect of iron-limitation on the PSII levels, a more intense effect occurs on the levels of the Cc_550_ heme protein subunit. The decay in the total Cc_550_ content under iron-deficient conditions is accompanied by a decrease in the amount of membrane-bound Cc_550_, but it is more remarkable that the decline in the Cc_550_ content is also accompanied by a drastic decay in the content of the PsbO subunit associated with the membrane, and therefore bound to the PSII core.

**Figure 3:**
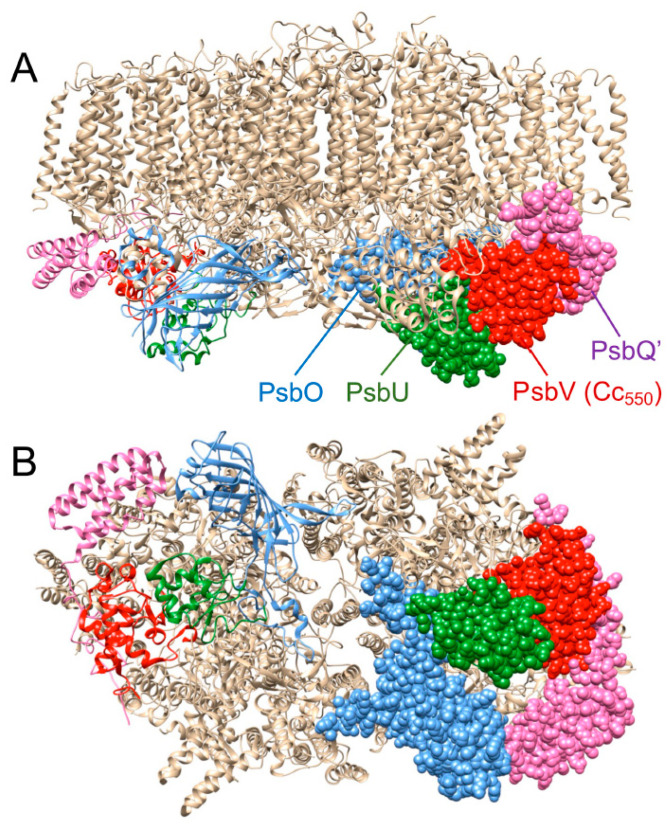
Photosystem II core of the diatom *C. gracilis*, PDB code, 6jlu [17]. (**A**) Membrane lateral view; (**B**) bottom luminal view (90° rotation). The main extrinsic subunits are highlighted: PsbO (blue), PsbV/cytochrome *c*_550_ (red), PsbU (green), PsbQ′ (purple). The Figure was generated with the UCSF Chimera program (version 1.16; http://www.rbvi.ucsf.edu/chimera/).

In addition to Cc_550_, PSII contains two other iron components: Cb_559_ and the non-heme iron cofactor [11], and iron deficiency, could also disturb PSII activity by affecting the levels of both components. However, the presence of Cb_559_ is essential for PSII function, as it is strictly required for PSII assembly [12], which could perhaps explain the observed decrease in PSII content under iron deficiency. On the other hand, the lack of the non-heme iron could result in the inhibition of the electron flow between Q_A_ and Q_B_ without affecting Q_A_ reduction [13,14]. However, as noted above, TL experiments indicate that iron limitation does not promote a significant blocking of ET from Q_A_ to Q_B_. Furthermore, a general decrease in PSII activity under iron-deficient conditions may be attributed to a reduced number of functional PSII complexes in cells [13], but also to the partial blocking of ET between PSII and PSI and the over-reduction of the PQ pool, due to a decrease in the levels of both the Cb_6_f complex and the Cc_6_ carrier [7,9,40]. An over-reduction of the PQ pool may induce a photoinhibition process on the acceptor side, which can decrease PSII activity [41]. However, beyond the effects due to the limitation in the electron flow, a specific effect of the lack of Cc_550_ was demonstrated in cyanobacteria, both in vitro and in Δ*psbV* mutants in vivo [27,29,31]. These works showed that the absence of Cc_550,_ in fact, affects PSII activity, but its presence is not strictly required, indicating an accessory role of Cc_550_ in the stabilization and functioning of the Mn_4_CaO_5_ cluster [27,29,31]. The specific effect of the lack of Cc_550_ on PSII activity can be explained by its role in stabilizing the water-splitting system and, in particular, in the binding of the other extrinsic subunits. The PSII of diatoms has an extra extrinsic protein, Psb31, in addition to the other four subunits also present in red algae: PsbO, PsbU, PsbQ′ and PsbV/Cc_550_ [16,26]. However, the recent crystal structure of the PSII from the diatom *C. gracilis* [17] (Figure 3) confirmed an overall structure similar to that of cyanobacterial PSII, including the position of Cc_550_ in the complex and its direct binding to the PSII core, in a manner essentially independent of the other extrinsic proteins. Thus, PsbV/Cc_550_ interacts with both PsbU and PsbQ′, which in turn establish contact with PsbO (Figure 3) [17]. In addition, it was reported that in Cc_550_ mutants in cyanobacteria, the weakening of the binding of PsbV/Cc_550_ to PSII not only promoted low levels of oxygen-evolving activity, but also weakened the binding of the extrinsic proteins PsbO and PsbU to PSII [29], the three proteins forming a protective cap that covers the catalytic core of PSII [42]. In these Cc_550_ mutants, the observed decrease in PSII activity was not caused by a lower amount of PSII core centers, but by the impairment of the water-splitting system due to the lack of the extrinsic subunits. Thus, the conclusion was that the loss of PsbV/Cc_550_, and the subsequent weakening of PsbO and PsbU binding, resulted in the incomplete assembly of fully functional PSII complexes and, consequently, a decreased water-splitting activity [29]. A similar situation would likely occur in *P. tricornutum* under strong iron limitations, in which an insufficient amount of Cc_550_, relative to the total amount of PSII core complexes, could specifically affect PSII activity, not only because of the direct role of Cc_550_ in the water-splitting function, but also by its effect on the binding of other equally required extrinsic subunits.

## 4. Materials and Methods

### 4.1. Cell Growth

Cells from the coastal pennate diatom *P. tricornutum* CCAP 1055/1 were grown as previously described [10] in Erlenmeyer flasks containing modified artificial seawater (ASW) medium [43,44] in a rotatory shaker (50 rpm) at 20 °C, with regular transfer into fresh media. The cultures were illuminated by LED white light (4500 K) lamps giving an intensity of 20 µmol m^−2^ s^−1^ (T8-150MWBL LED lamps; Wellmax) under a light/dark cycle of 16/8 h. Ultrapure Milli-Q^®^ water (Merck-Millipore, Darmstadt, Germany; resistivity of ≈18 MΩ.cm) was used to prepare the culture media. All the material used for the iron-deplete cultures was thoroughly cleaned with 3.7% HCl. To study the effects of iron deficiency, cells from 7-day-old cultures were pelleted, suspended and grown in modified ASW medium, with 10 µM iron (iron-replete cultures) or with 0.1 or 0.04 µM (iron-deplete cultures) [45]. The cells were transferred into fresh media after 7 days and grown for a further 15 days. The cell density of the cultures was measured at 730 nm, and cell concentrations were determined by cell counting with a Neubauer improved hemocytometer (Marienfeld-Superior). For most of the experiments, the initial optical density at 730 nm of the cultures was 0.1 (1.5 × 10^6^ cells mL^−1^). Cell specific growth rate (µ, day^−1^) was calculated after 15 days of culture growth as: [µ = ln (N_t_/N_0_)/Δt] where N_t_ and N_0_ are the final and initial cell concentration, respectively, and Δt is the days of growth [46]. Growth cell parameters were obtained from at least ten independent experiments.

### 4.2. Protein Analysis Methods 

Protein extraction from 25–50 mL of *P. tricornutum* cultures was carried out by following the protocol described in [10]. Total protein contents were quantified by a modification of the Lowry method [47,48] by using a standard curve from known concentrations of bovine serum albumin. 

The Cc_6_ and Cc_550_ content in *P. tricornutum* cells was measured following the methods previously described [10,22]. Cells were broken by three cycles of French press disruption (20,000 psi), followed by differential clarification and precipitation with ammonium sulphate (55% and 100% for Cc_6_; 30% and 60% for Cc_550_). Cc_6_ and Cc_550_ concentrations were determined by differential absorbance measurements in the final solutions between the fully reduced (sodium ascorbate for Cc_6_; sodium dithionite for Cc_550_) and fully oxidized (potassium ferricyanide for Cc_6_; sodium ascorbate for Cc_550_), using a differential extinction coefficient of 15 mM^−1^ cm^−1^ at 552 (Cc_6_) or 550 (Cc_550_) nm [49,50]. 

To estimate the amount of the PSII subunits Cc_550_ (PsbV), D1 and PsbO associated with membrane, cells from 75 mL cultures were collected and resuspended in 25 mL of 50 mM MES, pH 6.5, buffer supplemented with 10 mM MgCl_2_, 1 M betaine, protease inhibitors (PMSF, benzamidine and aminocaproic acid) and DNase. Cells were then disrupted by three French press cycles at 7000 psi. Unbroken cells were separated by centrifugation at 5000× *g* for 5 min and supernatants were centrifuged at 170,000× *g* for 25 min. The resultant pellets were resuspended in the same buffer to obtain the membrane extracts. The absence of Cc_6_ was used as a quality control for these final extracts.

For Western-blot analyses, polyclonal antibodies raised against *P. tricornutum* Cc_6_ and Cc_550_ were already available in the lab [9,22]. Antibodies against D1, PsbO and the RubisCO large subunit were purchased from Agrisera (Vännäs, Sweden). Protein extracts (2.5–20 µg of total protein) were resolved on 15% (*w*/*v*) SDS-polyacrylamide gel electrophoresis as previously described [9]. Protein transfer to nitrocellulose membranes, as well as membrane treatment and visualization, were carried out as described in Castell et al. [10]. Ponceau total protein staining was used as the loading control in the immunoblottings. In some cases, Western-blot bands from at least three different immunoblottings were quantified using the Quantity One^®^ 1-D analysis software (Bio-Rad).

### 4.3. Photosynthetic Measurements

Standard thermoluminescence (TL) glow curves of *P. tricornutum* cells suspensions were obtained using a home-built apparatus designed by Dr. Jean-Marc Ducruet for luminescence detection from 1 °C to 80 °C [9,51]. TL measurements were carried out as described in Roncel et al. [9] and Castell et al. [10] in the absence or presence of DCMU 20 µM. Data acquisition, signal analysis and graphical simulation were performed as previously described [52,53,54]. TL parameters were obtained from six independent experiments.

To study PSII global activity, cells were collected by centrifugation and resuspended in fresh ASW medium to a similar cell density. Oxygen intake or evolution was then determined in 2.5 mL samples from three independent experiments by using a Clark-type oxygen electrode (Oxylab^+^, Hansatech) at 20 °C, both in the dark and under illumination (176 µmol m^−2^ s^−1^), to establish the net photosynthetic activity per cell using DCBQ as the electron acceptor. 

The chlorophyll *a* fluorescence of PSII from intact cells was determined at room temperature using a pulse-amplitude modulation fluorometer (DUAL-PAM-100, Walz, Effeltrich, Germany). Photosynthetic parameters were obtained from cell suspensions with a cellular concentration of 1.1 × 10^8^ cells mL^−1^ in five independent experiments, carried out as described in Roncel et al. [9]. The relative PSII content in intact cells was estimated from the total yield of variable chlorophyll *a* fluorescence, or *F*_v_ = *F*_m_ − *F*_0_ [37,38]. *F*_o_ (minimum fluorescence in dark-adapted state) was measured when Q_A_ was fully oxidized by incubating cells in darkness with *p*-benzoquinone and ferricyanide. *F*_m_ (maximum fluorescence) was determined when Q_A_ was fully reduced by illuminating cells in the presence of DCMU and hydroxylamine. The samples were incubated in darkness for 30 min, followed by an incubation step with 0.3 mM *p*-benzoquinone and 1 mM ferricyanide for 5 min. DCMU was then added up to a concentration of 40 µM and incubated for 1 min in darkness. Next, hydroxylamine was added to a concentration of 20 mM from a freshly prepared 1 M stock solution (pH 6.5). The fluorescence measuring light was turned on 20 s after the last addition to record *F*_0_. Later, *F*_m_ was determined after one single saturating flash. The maximum quantum yield of PSII (*F*_v_/*F*_m_), as a measurement of the PSII/LHC integrity, was also determined as previously described [9].

### 4.4. Statistical Significance Level

The significance of the results from two data sets was analyzed using a Two Sample *t*-Test calculator tool (https://www.statskingdom.com (accessed on 4 October 2022). Data inputs were: means, standard deviations and N (number of measurements per group). Data were considered significantly different for a *p*-value < 0.05. 

## Figures and Tables

**Table 1:** Growth and photosynthetic parameters of *P. tricornutum* cells under different conditions of iron availability.

Parameter (after 15 Days of Growth)	10 µM Fe	0.1 µM Fe	0.04 µM Fe
Specific growth rate (µ, day^−1^)	0.252 ± 0.002 (100%)	0.204 ± 0.009 (80.9%)	0.190 ± 0.011 (75.4%)
Net respiration rate per 10^6^ cells (nmol O_2_ h^−1^)	−0.57 ± 0.02 (100%)	−0.93 ± 0.01 (163.1%)	−0.82 ± 0.01 (142.6%)
Net photosynthetic rate per 10^6^ cells (nmol O_2_ h^−1^)	6.51 ± 0.03 (100%)	3.16 ± 0.05 (48.5%)	2.72 ± 0.04 (41.5%)
TL intensity (a.u.)	340.9 ± 20.5 (100%)	206.7 ± 17.3 (60.6%)	161.2 ± 9.4 (47.3%)
Relative PSII content (*F_m_ − F*_0_)	0.142 ± 0.037 (100%)	0.084 ± 0.017 (59.1%)	0.079 ± 0.021 (55.4%)
Maximum quantum yield of PSII (*F*_v_/*F*_m_)	0.621 ± 0.016 (100%)	0.463 ± 0.050 (74.5%)	0.394 ± 0.014 (63.4%)

## Data Availability

Not applicable.

## Supplementary Materials

The following supporting information can be downloaded at: https://www.mdpi.com/article/10.3390/ijms232012138/s1.
