# Supplementary material for: Iron Deficiency Promotes the Lack of Photosynthetic Cytochrome c550 and Affects the Binding of the Luminal Extrinsic Subunits to Photosystem II in the Diatom Phaeodactylum tricornutum
Source: Int J Mol Sci. 2022 Oct 12;23(20):12138. doi: 10.3390/ijms232012138 (PMC9603157; doi:10.3390/ijms232012138)
Supplement: Supplementary file 1 [file ijms-23-12138-s001.zip › ijms-1946906-supplementary.pdf]

Supplementary Material:

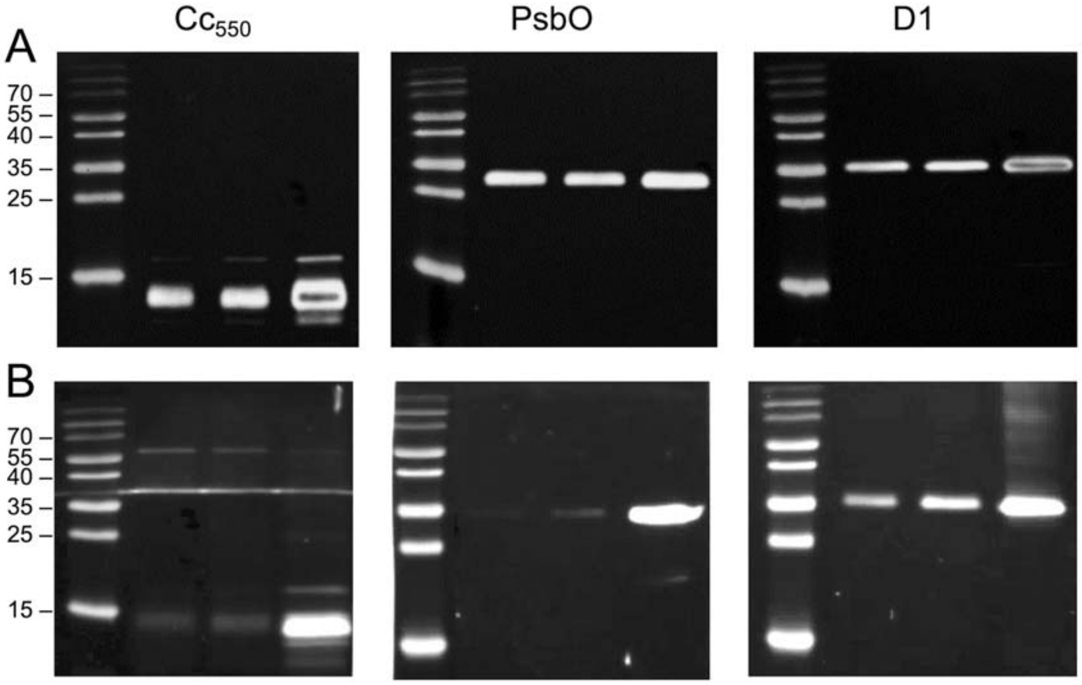

Figure S1. Representative Western-blots of the Cc_{550}, PsbO and D1 photosynthetic proteins shown in Figure 1. (A) Samples of whole cells. (B) Membrane extracts. See Figure 1 for further details. Numbers on the left are the MW values (kDa) corresponding to the molecular weight standard kit (left column of the Western-blots).
